# Research on drought stress in *Medicago sativa L.* from 1998 to 2023: a bibliometric analysis

**DOI:** 10.3389/fpls.2024.1406256

**Published:** 2024-05-30

**Authors:** Zijun Zhou, Junqin Li, Yang Gao, Xiangtao Wang, Rui Wang, Haiyan Huang, Yu Zhang, Lili Zhao, Puchang Wang

**Affiliations:** ^1^ School of Life Sciences, Guizhou Normal University, Guiyang, Guizhou, China; ^2^ School of Karst Science, Guizhou Normal University, Guiyang, Guizhou, China; ^3^ College of Animal Science, Guizhou University, Guiyang, Guizhou, China

**Keywords:** alfalfa, drought tolerance, response mechanism, yield, bibliometrics, research trends

## Abstract

Alfalfa (*Medicago sativa L*.) is one of the most important forage crops in the world. Drought is recognized as a major challenge limiting alfalfa production and threatening food security. Although some literature reviews have been conducted in this area, bibliometric reviews based on large amounts of published data are still lacking. In this paper, a bibliometric analysis of alfalfa drought stress from 1998–2023 was conducted using the Web of Science Core Collection database in order to assess global trends in alfalfa drought stress research and to provide new directions for future research. The results showed that the annual publication output maintained an increase in most years, with China and the United States contributing significantly to the field. Most of the journals published are specialized journals in botany, environmental science, soil science and crop science, as well as related agribusiness journals. “plant growth” and “yield” were the most frequently used keywords, reflecting the important purpose of research in this field. And two main research directions were identified: research on drought response mechanism of alfalfa and exploration of drought-resistant technology. In addition, physiological, biochemical, and molecular responses of drought tolerance and high yield in alfalfa, transgenics, and microbial fertilizer research have been hot research topics in recent years and may continue in the future. The ultimate goal of this paper is to provide a foundational reference for future research on alfalfa’s drought resistance and yield optimization mechanisms, thereby enhancing the crop’s application in agricultural production.

## 1 Introduction

Recent years have seen global climate change inducing higher temperatures, which in turn has increased potential evapotranspiration and exacerbated drought conditions, particularly in semi-arid regions (Muluneh et al., 2014). Soil moisture, a critical factor for crop growth, is significantly compromised in these arid areas. The heightened water consumption by crops further aggravates soil drought, consequently diminishing crop productivity (Brookshire and Weaver, 2015). Drought is identified as a primary cause of reduced crop yields (Araya et al., 2012). Plant drought tolerance is described as the capacity of plants to sustain growth under less than optimal water supply conditions (Luo, 2010). Adaptation mechanisms to combat drought include osmotic regulation, production of protective metabolites, proteins, and systems for scavenging reactive oxygen species (ROS) (Ma et al., 2016). When soil water content is limited, plants experience alterations in their metabolism across developmental, physiological, and molecular levels. These changes lead to decreased growth and photosynthesis rates, modifications in photosynthetic proton and electron transport, and reductions in carbon oxidation cycling and photosynthetic carbon assimilation (Cornic, 2002; Zivcak et al., 2014; Putnam, 2021). Concurrently, drought stress can lead to a loss of cellular turgor and lower water content in cells (Hernandez-Santana et al., 2021), further impeding plant growth and accumulation of dry mass (Ogunkanmi et al., 2021; Wan et al., 2022). Notably, drought stress is a significant environmental challenge impeding the growth and yield of alfalfa globally (Diatta et al., 2021), characterized by its unpredictability and considerable adverse impacts on global crop production (Golldack et al., 2014; Anjum et al., 2017; Hussain et al., 2018). Water deficits inflict harm on plants by disrupting various physiological processes, such as carbon assimilation, cellular hydration, increased oxidative damage, and leaf gas exchange, culminating in lower yields (Chowdhury et al., 2016; Hussain et al., 2018). In the face of global warming, the development of drought tolerance in crops has become an increasingly pressing issue.

Alfalfa (*Medicago sativa L.*), often hailed as the “queen of forages,” is a venerable wild plant originally found in the Mediterranean mountain forests of southwestern Asia. The term “alfalfa” stems from the Arabic “Al-Fasfasa,” meaning “the father of all plants” (Lacefield et al., 1979). Renowned globally as a vital perennial legume forage (Lamb et al., 2006; Veronesi et al., 2010; Annicchiarico et al., 2014), alfalfa is celebrated for its high yield, superior quality, and rich protein content. Notably, it thrives on marginal lands (Brawley and Mathes, 1990), cementing its status as one of the most sought-after forage legumes due to its nutritional value and productivity (Basigalup et al., 2014). Alfalfa enhances soil structure through its deep-rooting system and leverages its symbiotic capability for biological nitrogen fixation with rhizobacteria (Carlsson and Huss-Danell, 2003), thereby boosting nitrogen availability for subsequent crops (Basigalup et al., 2014). Rhizosphere bacteria play a key role in improving the adaptability of alfalfa to drought stress (Fan et al., 2023). Exceptionally adaptable, alfalfa flourishes in various environments, particularly under drought conditions (Annicchiarico, 2007; Huang et al., 2018). Its deep root system contributes to its relative drought tolerance, especially beneficial in semi-arid regions (Ma et al., 2021). With numerous research papers on alfalfa under water stress published globally, effectively synthesizing this vast array of information is essential for researchers to gain a comprehensive understanding of the current research trends and future directions in a timely manner.

Bibliometric analysis employs statistical and visual techniques to dissect the complex characteristics of a body of published literature (Broadus, 1987; Okubo, 1997; Van Raan et al., 2010). This approach enables researchers to swiftly pinpoint relevant topics and directions amidst a vast array of literature, clarifying key information, contextualizing findings, and identifying the most active research frontiers and trends. Unlike traditional literature reviews and meta-analyses, bibliometric analysis provides a more holistic grasp of the current state, forefront, and potential future trends of a specific research field. To date, only a limited number of scholars have utilized bibliometric methods to visualize and analyze research on alfalfa drought tolerance. Thus, this study adopts a bibliometric approach, using tools such as VOSviewer and R, to dissect and elucidate the knowledge structure within alfalfa drought tolerance research. Our objective is to facilitate rapid access to the core research content and prevailing topics in this field.

## 2 Materials and methods

### 2.1 Data sources and screening

**Figure 1:**
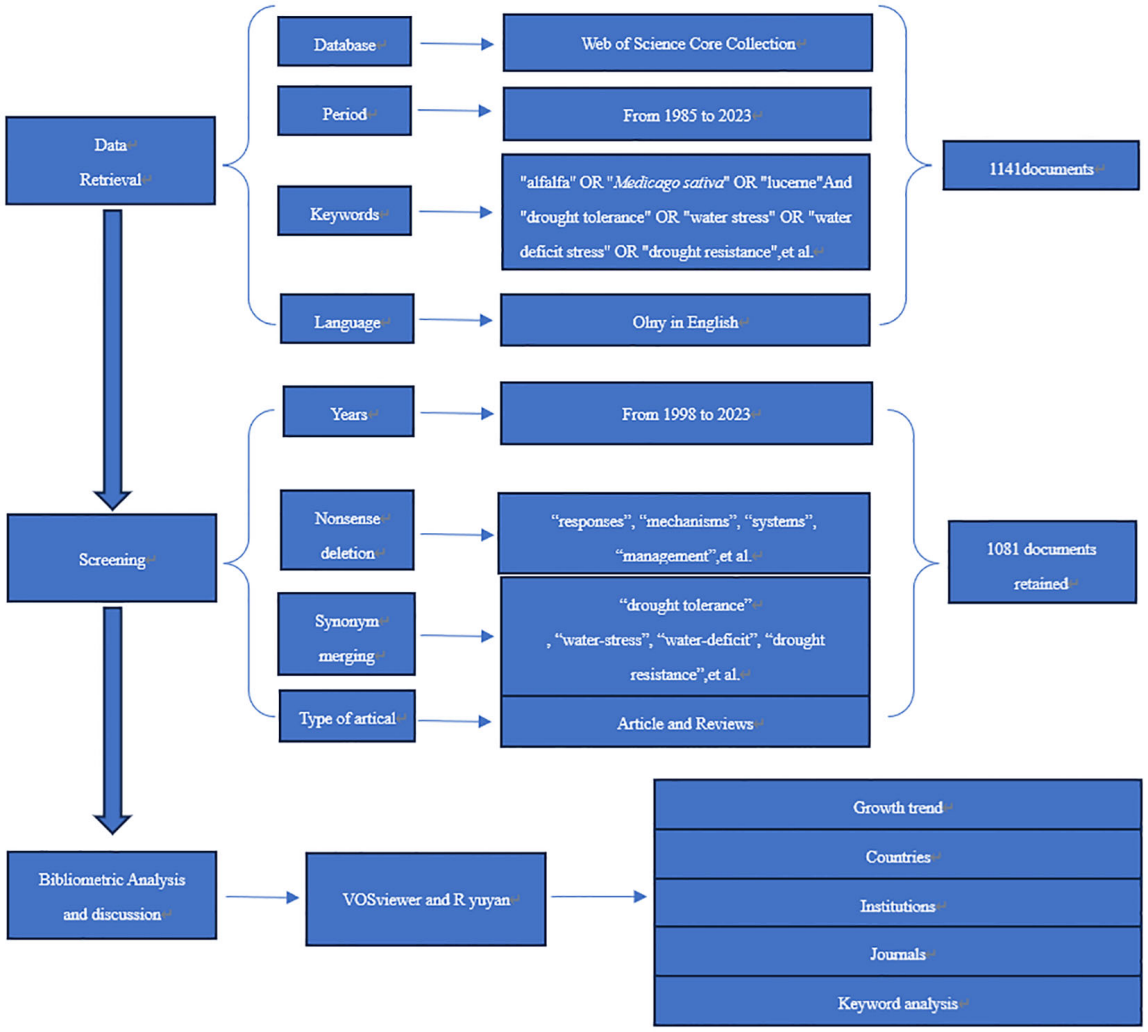
The research flowchart of present study.

On November 23, 2023, we employed specific keywords to retrieve literature from the Web of Science Core Collection (WSCC) database at the University of Shanghai for Science and Technology (USST) Library. The search terms included combinations of “alfalfa or *Medicago sativa* or lucerne” with “drought tolerance or water stress or water deficit stress or drought-tolerant or drought persistence, et al.” Recognizing that the earliest alfalfa-related literature in the Core Collection dates back to 1998, we selected a corpus of 1,081 articles and reviews covering the period from 1998 to 2023. For data export, we chose the “Full Record and Cited References” option, enabling the exportation of 500 articles at a time. Consequently, all literature was exported in three batches. The exported “text” files were initially processed using Co-Occurrence 9.9 (COOC) and subsequently converted into “excel” files for further analysis. To enhance the accuracy of our analysis, irrelevant keywords were omitted, and synonyms were consolidated. The selection and flow of this study are depicted in **Figure 1**.

### 2.2 Data visualization and analysis

The data for analysis, encompassing various metrics such as the number of publications by countries/regions, annual publications, journal contributions, citations, etc., were sourced from the WOS core dataset (Tan et al., 2021). We commenced with metric frequency analysis and interaction analysis to gain a preliminary understanding of the major contributing countries, institutions, and journals, as well as the extent of collaborative ties. This process was primarily conducted using VOSviewer (version 1.6.11) from the bibliometrics software suite, and R (version 4.3.0) for bibliometric analysis and visualization. The key operation involved using VOSviewer to extract information regarding the top 20 countries, institutions, and journals by publication count, the top 30 most cited documents, and the top 20 most recurrent Keywords Plus. Additionally, we created knowledge domain maps depicting co-authorship among countries, institutional coupling, journal co-citations, and keyword co-occurrences. In R, we generated knowledge domain maps for thematic keywords and trend topics. Given that author keywords tend to be highly subjective and lack statistical uniformity (Qin et al., 2021), we opted for Keywords Plus for its superior accuracy, authoritative nature, and statistical relevance for data analysis and visualization in this study.

## 3 Results

### 3.1 The publication trends

**Figure 2:**
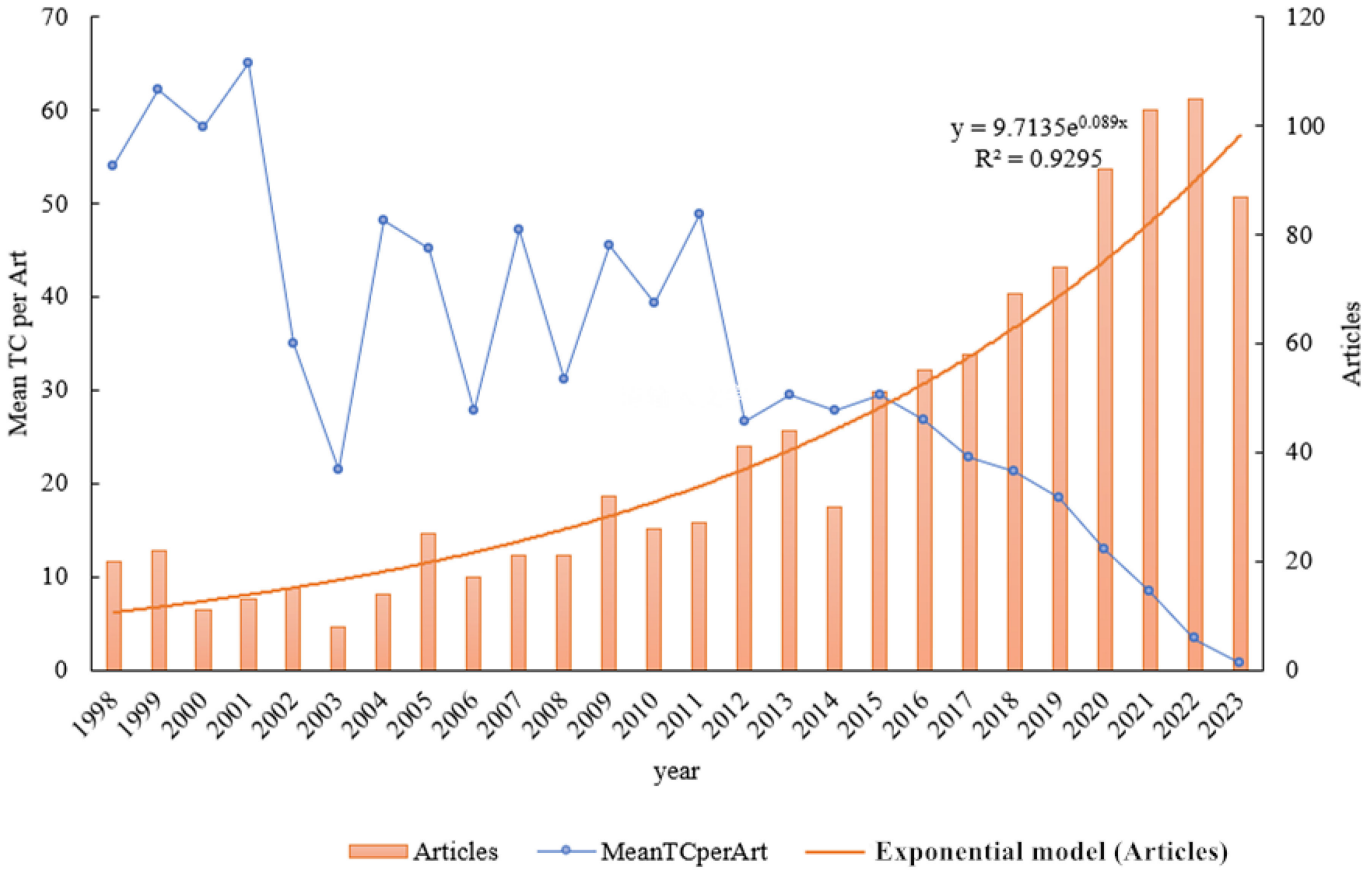
Temporal evolution of publications on drought stress in *Medicago sativa* from 1998 to 2023. Mean TC per Art -mean total citation per article.

The temporal progression of publications pertaining to alfalfa drought research over the period from 1998 to 2023 is depicted in **Figure 2**. An examination of the figure shows a fluctuating increase in the number of related publications. However, there is an observable downward trend in the average total number of citations per article. This decline is likely attributable to factors such as the varying quality of article content and the timing of their publication. The coefficient of determination, R², is used to quantify the fit of the trend line representing changes in publication numbers over this 26-year span. In this study, R² is calculated to be 0.949. This high value suggests a strong correlation and indicates that the trend line closely aligns with the actual data, reflecting a significant and consistent increase in the volume of publications on alfalfa drought research over the studied period.

### 3.2 Countries and institutions distribution for the literature

**Figure 3:**
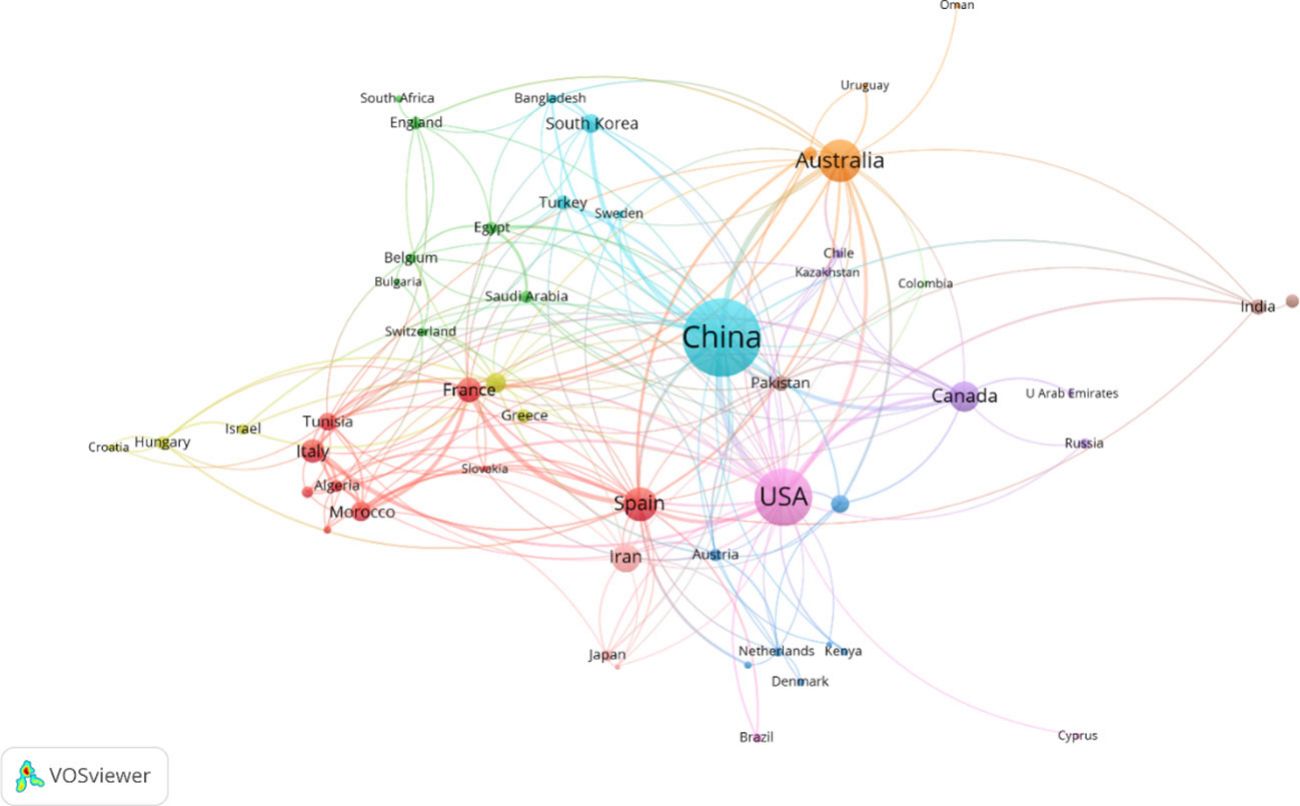
Mapping knowledge domain of co-authoring countries/regions on drought stress in *Medicago sativa* (frequency ≥ 2).

The distribution and collaboration dynamics among countries and regions engaged in alfalfa drought research are elucidated in **Figure 3**. This figure, coupled with the data in **Supplementary Table S1**, displays the number of articles published by each of the 53 participating countries and the nature of their collaborative relationships. China emerges as the most prolific contributor, with the largest node representing its 385 published articles. Furthermore, China has established collaborative ties with 26 countries, showcasing its closest collaboration with the United States (indicated by the highest Total Link Strength, TLS), followed by Australia. The United States, represented as the second largest node, has published 213 articles and has fostered collaborations with 39 countries. Overall, while China leads in article contribution, the United States demonstrates a broader spectrum of international collaborations. This suggests that China, despite its significant contributions, could benefit from enhancing its international cooperative efforts in this research domain.

**Figure 4:**
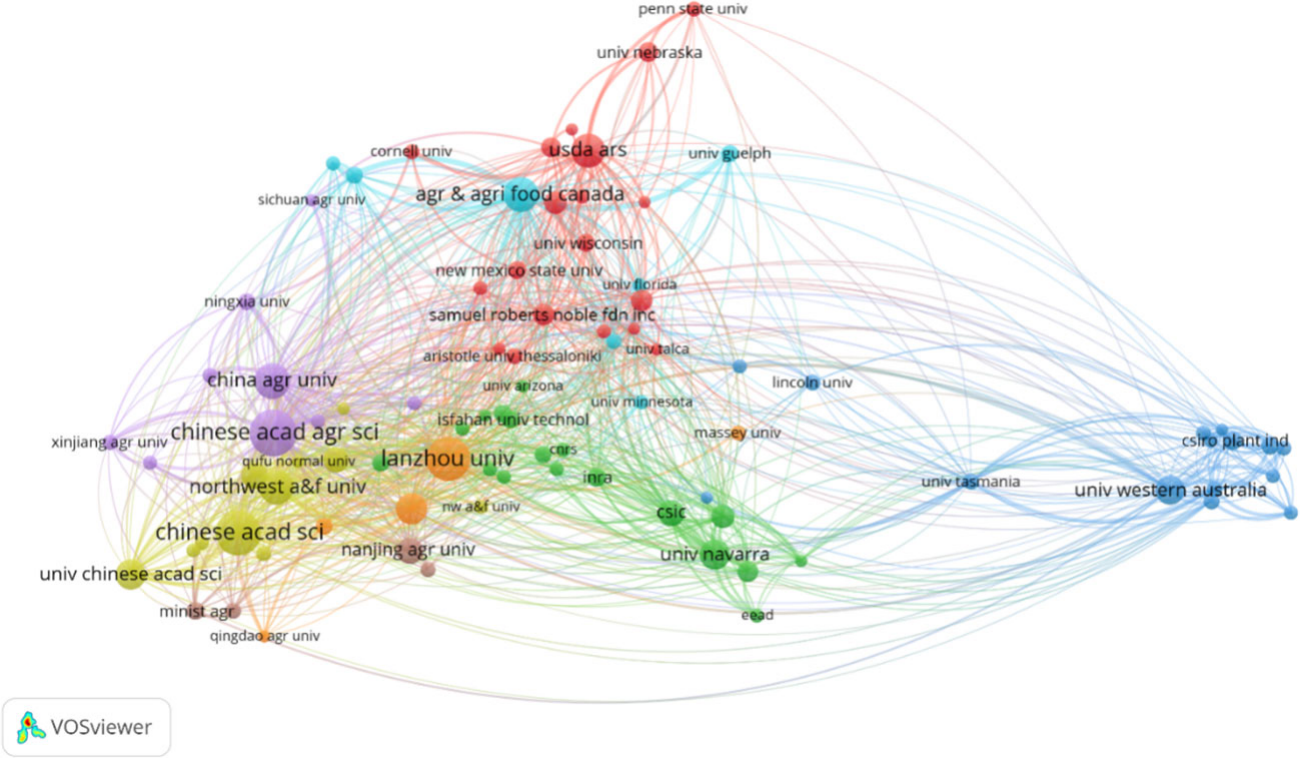
Bibliographic coupling analysis of institutions on drought stress in *Medicago sativa* (frequency ≥ 5).

The analysis of institutional collaboration offers insights into organizational contributions and inter-institutional interactions within this research topic. **Figure 4**, integrated with the details in **Supplementary Table S2**, presents an inter-institutional literature coupling analysis through scientific knowledge mapping. The Chinese Academy of Agricultural Sciences (CAAS) stands out as not only the most productive institution, with 64 publications, but also as a central node in the field, exhibiting the widest range of domain relationships (with the largest TLS). Following CAAS, the Chinese Academy of Sciences (CAS) with 59 publications and Lanzhou University (LU) with 56, both based in China, play significant roles in the scholarly network. The predominance of Chinese institutions in alfalfa drought research, as depicted in **Figure 4**, aligns with the trends observed in the country-based analysis in **Figure 3**. This highlights China’s leading position in the research of drought stress in *Medicago sativa*.

### 3.3 Main journals and most impacting papers

**Figure 5:**
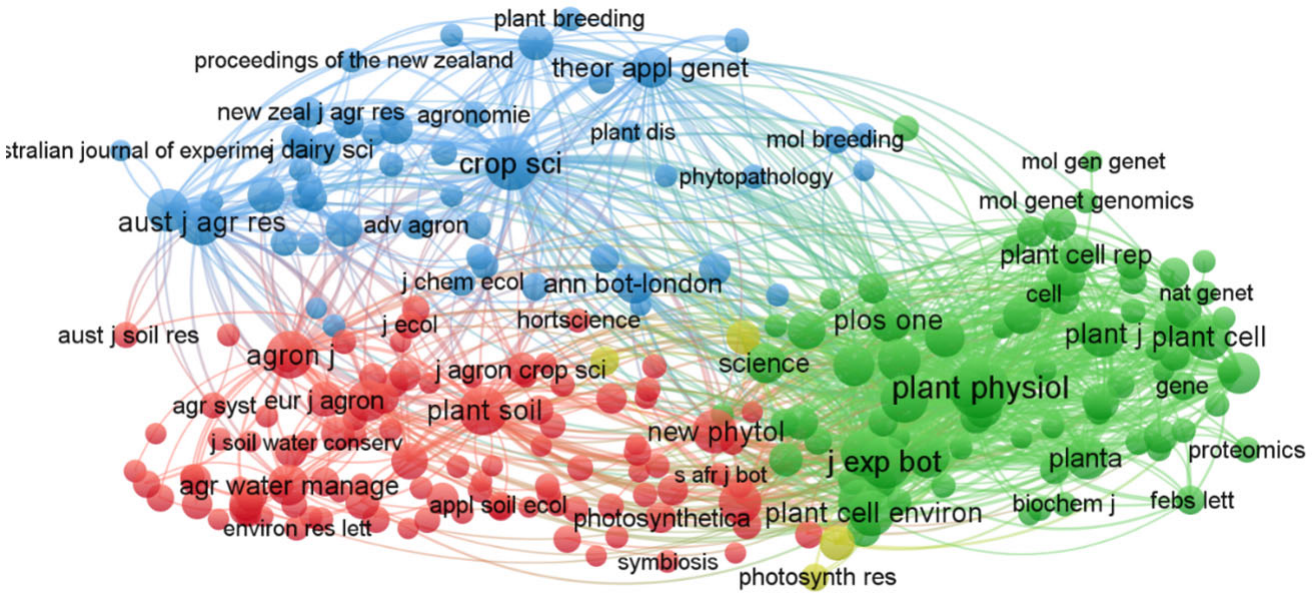
Mapped knowledge domains for journal co-citation on drought stress in *Medicago sativa* (frequency ≥ 30).

In this study, co-citation analysis was specifically applied to source journals to identify the core journals and foundational knowledge in alfalfa drought research. **Figure 5**, along with **Supplementary Table S3**, illustrates the knowledge map of 261 journal co-citations, shedding light on the scientific interrelationships among these journals. The largest node, representing Plant Physiology (plant physiol), with 2,268 citations, emerges as the most cited journal in this research area. Following closely are the Journal of Experimental Botany (j exp bot) with 1,313 citations and Crop Science (crop sci) with 1,272 citations. The thickest line between Plant Physiology and the Journal of Experimental Botany denotes the strongest co-citation link, indicating that articles from these two journals are frequently cited together. Similarly, notable connections are observed between Plant Journal (plant j) and Plant Cell. These four journals, especially Plant Physiology, hold significant citation counts, marking them as key publications in the field of alfalfa drought research. The division into four distinct clusters is evident: the green cluster, led by Plant Physiology, focuses on plant material metabolism, growth, and environmental interactions; the red cluster, anchored by Plant and Soil (plant soil), delves into plant biology and soil science interactions; the blue cluster, centered on Crop Science, investigates crop genetics and breeding; and the yellow cluster, headed by the Journal of Experimental Botany, publishes work on sustainable food, fuel, and renewable materials production. To summarize, alfalfa drought research predominantly features in specialized journals across plant science, environmental science, soil science, and crop science, as well as related agribusiness journals.

From the dataset, a total of 3,821 authors contributed to 1,081 articles on alfalfa drought tolerance. The top 30 cited articles were selected for a focused bibliometric analysis to determine their influence on the development of alfalfa drought research. The most cited article, with 312 citations, is by Zhang, JY et al., published in Plant Journal in 2005. The next two highly cited works are by Aranjuelo, Iker et al., in the Journal of Experimental Botany (2011), and by Antoniou, Chrystalla et al., in the Journal of Pineal Research (2011). These articles are highly recognized in the field, as evidenced by their citations. The analysis indicates that alfalfa drought research primarily involves collaborations among domestic institutions, highlighting a need for enhanced international collaboration.

According to the content of these 30 papers (**Table 1**), the research involving the field of drought stress in alfalfa was found to include: (1) Studies on the morphology of alfalfa leaves, epidermal wax content,composition or structure, and alkane content under drought stress; (2) Adaptation to drought by photosynthesis, osmotic regulation, antioxidant defense, and administration of exogenous hormones to the plant; (3) Excavation and identification of drought-resistant functional genes, and transgenic alfalfa research; (4) Variety selection and molecular breeding using molecular marker technology, etc.; (5) Irrigation, planting time adjustment, nutrient management and seed initiation in alfalfa. In general, it is morphology, physiology and biochemistry, molecular response mechanism research and drought-resistant breeding and cultivation technology research.

**Table 1:** Top 30 publications with the most citations on drought stress in *Medicago sativa*.

Rank	Title	Journal	Year	TC^c^	IF	CN	ON
(1)	Overexpression of *WXP1*, a putative *Medicago truncatula* AP2 domain-containing transcription factor gene, increases cuticular wax accumulation and enhances drought tolerance in transgenic alfalfa (*Medicago sativa*)	Plant Journal	2005	312	7.2	1	1
(2)	Plant physiology and proteomics reveals the leaf response to drought in alfalfa (*Medicago sativa L.*)	Journal Of Experimental Botany	2011	197	6.9	2	3
(3)	Melatonin systemically ameliorates drought stress-induced damage in *Medicago sativa* plants by modulating nitro-oxidative homeostasis and proline metabolism	Journal Of Pineal Research	2017	195	10.3	1	2
(4)	The response of carbon metabolism and antioxidant defenses of alfalfa nodules to drought stress and to the subsequent recovery of plants	Plant Physiology	2007	160	7.4	2	3
(5)	MicroRNA156 improves drought stress tolerance in alfalfa (*Medicago sativa*) by silencing SPL13	Plant Science	2017	136	5.2	1	2
(6)	Biomass partitioning, morphology and water status of four alfalfa genotypes submitted to progressive drought and subsequent recovery	Journal Of Plant Physiology	2010	116	4.3	3	3
(7)	Effects of water stress on antioxidant enzymes of leaves and nodules of transgenic alfalfa overexpressing superoxide dismutases	Physiologia Plantarum	2002	111	6.4	2	3
(8)	System responses to long-term drought and re-watering of two contrasting alfalfa varieties	Plant Journal	2011	103	7.2	1	1
(9)	Response of alfalfa to putrescine treatment under drought stress	Biologia Plantarum	2006	96	1.5	1	3
(10)	Physiological and proteomic responses of contrasting alfalfa (*Medicago sativa L.*) varieties to PEG-Induced osmotic stress	Frontiers In Plant Science	2018	77	5.6	1	1
(11)	Soil water storage deficit of alfalfa (*Medicago sativa*) grasslands along ages in arid area (China)	Field Crops Research	2018	73	5.8	1	4
(12)	The interplay between *miR156/SPL13* and *DFR/WD40–1* regulate drought tolerance in alfalfa	BMC Plant Biology	2019	68	5.3	2	3
(13)	Application of sewage sludge improves growth, photosynthesis and antioxidant activities of nodulated alfalfa plants under drought conditions	Environmental And Experimental Botany	2010	67	5.7	1	2
(14)	Drought tolerance in alfalfa (*Medicago sativa L.*) varieties is associated with enhanced antioxidative protection and declined lipid peroxidation	Journal Of Plant Physiology	2019	65	4.3	1	1
(15)	Improved drought stress response in alfalfa plants nodulated by an IAA over-producing *Rhizobium Strain*	Frontiers In Microbiology	2017	54	5.2	2	4
(16)	Water use efficiency, transpiration and net CO_2_ exchange of four alfalfa genotypes submitted to progressive drought and subsequent recovery	Environmental And Experimental Botany	2011	54	5.7	3	3
(17)	Exploring the potential of nitric oxide and hydrogen sulfide (NOSH)-releasing synthetic compounds as novel priming agents against drought stress in *Medicago sativa* plants	Biomolecules	2020	53	5.5	2	3
(18)	Comparative physiological and transcriptional analyses of two contrasting drought tolerant alfalfa varieties	Frontiers In Plant Science	2016	52	5.6	2	2
(19)	Identification of loci associated with drought resistance traits in heterozygous autotetraploid alfalfa (*medicago sativa L.*) using genome-wide association studies with genotyping by sequencing	PLoS One	2015	51	3.7	1	2
(20)	Influence of arbuscular Mycorrhizae and *Rhizobium* on free polyamines and proline levels in water-stressed alfalfa	Journal Of Plant Physiology	1998	48	4.3	2	2
(21)	Influence of drought stress on afalfa yields and nutritional composition	PLoS One	2018	39	5.3	1	3
(22)	Maximizing productivity and water use efficiency of alfalfa under precise subsurface drip irrigation in arid regions	Irrigation And Drainage	2013	39	1.9	2	3
(23)	Physiological and morphological traits associated with adaptation of lucerne (*Medicago sativa*) to severely drought-stressed and to irrigated environments	Annals Of Applied Biology	2013	39	2.6	1	1
(24)	Transcriptome analysis of microRNA156 overexpression alfalfa roots under drought stress	Scientific Reports	2018	36	4.6	2	2
(25)	Physiological and biochemical changes in different drought-tolerant alfalfa (*Medicago sativa L.*) varieties under PEG-induced drought stress	Acta Physiologiae Plantarum	2018	36	2.6	1	2
(26)	Effects of water deficit on growth, nodulation and physiological and biochemical processes in *Medicago sativa*-rhizobia symbiotic association	Arid Land Research And Management	2016	36	1.4	2	3
(27)	Effects of engineered *sinorhizobium meliloti* on cytokinin synthesis and tolerance of alfalfa toextreme drought stress	Applied And Environmental Microbiology	2012	35	4.4	1	1
(28)	Seed osmopriming improves plant growth, nodulation, chlorophyll fluorescence and nutrient uptake in alfalfa (*Medicago sativa L.*) - rhizobia symbiosis under drought stress	Scientia Horticulturae	2016	34	4.3	1	3
(29)	Concerted changes in N and C primary metabolism in alfalfa (*Medicago sativa*) under water restriction	Journal Of Experimental Botany	2013	33	5.3	3	5
(30)	Leaf cuticular waxes and physiological parameters in alfalfa leaves as influenced by drought	Photosynthetica	2012	32	2.7	2	2

c, Total Citations; IF-Impact Factors; d, Avg. citations; e, Links; f, Total Link Strength; CN, Cooperative Nations Number; ON, Cooperative Organizations Number.

### 3.4 Research hotspots and evolution trend analysis

#### 3.4.1 Topic keywords map and hotspot evolutionary trend

The co-occurrence of keywords in literature offers a gateway to understanding the intrinsic connections, research hotspots, and predictive development trends in an academic field. **Table 2** shows the top 20 most frequently occurring keywords in the field of alfalfa drought tolerance research. Notably, “drought stress” and “*medicago-sativa*” are predominant, followed by “plant-growth” and “yield”. This pattern indicates the significant impact of drought on both the growth and yield of alfalfa, highlighting the importance of understanding the drought-plant growth-yield relationship as a foundational research area in this field. The presence of “gene-expression” as a key keyword reflects the focus on genetic regulation as a means for alfalfa to combat drought stress. The inclusion of “arabidopsis” suggests its use as a model organism for gene function, genetics research, and related areas, pointing towards transgenic research in alfalfa as a current research hotspot. Based on these keywords, the current research in alfalfa drought tolerance encompasses (1) Enhancing plant growth and crop yield, and (2) Transgenic research, physiological and biochemical response mechanisms, and nutrient management as key research hotspots.

**Table 2:** Top 20 most frequently used keywords on drought stress in *Medicago sativa*.

Rank	Keyword Plus	Cluster	Occurrences	Links	TLS^f^
1	drought stress	1	555	302	2416
2	*medicago-sativa*	2	317	272	1425
3	plant-growth	9	203	194	911
4	yield	2	190	187	778
5	gene-expression	1	172	146	828
6	arabidopsis	1	105	108	516
7	nitrogen-fixation	7	65	105	341
8	abscisic-acid	1	63	104	336
9	oxidative stress	11	53	86	265
10	proline	1	50	85	268
11	photosynthesis	6	49	84	272
12	CO_2_	5	48	88	254
13	water-use efficiency	3	47	91	213
14	n-fertilization	6	45	104	218
15	leaf	10	42	85	203
16	gas-exchange	1	41	76	184
17	transcription factors	7	38	78	210
18	root	8	37	74	173
19	lipid-peroxidation	1	36	63	185
20	chlorophyll	8	28	61	155

^f^, Total Link Strength.

**Figure 6:**
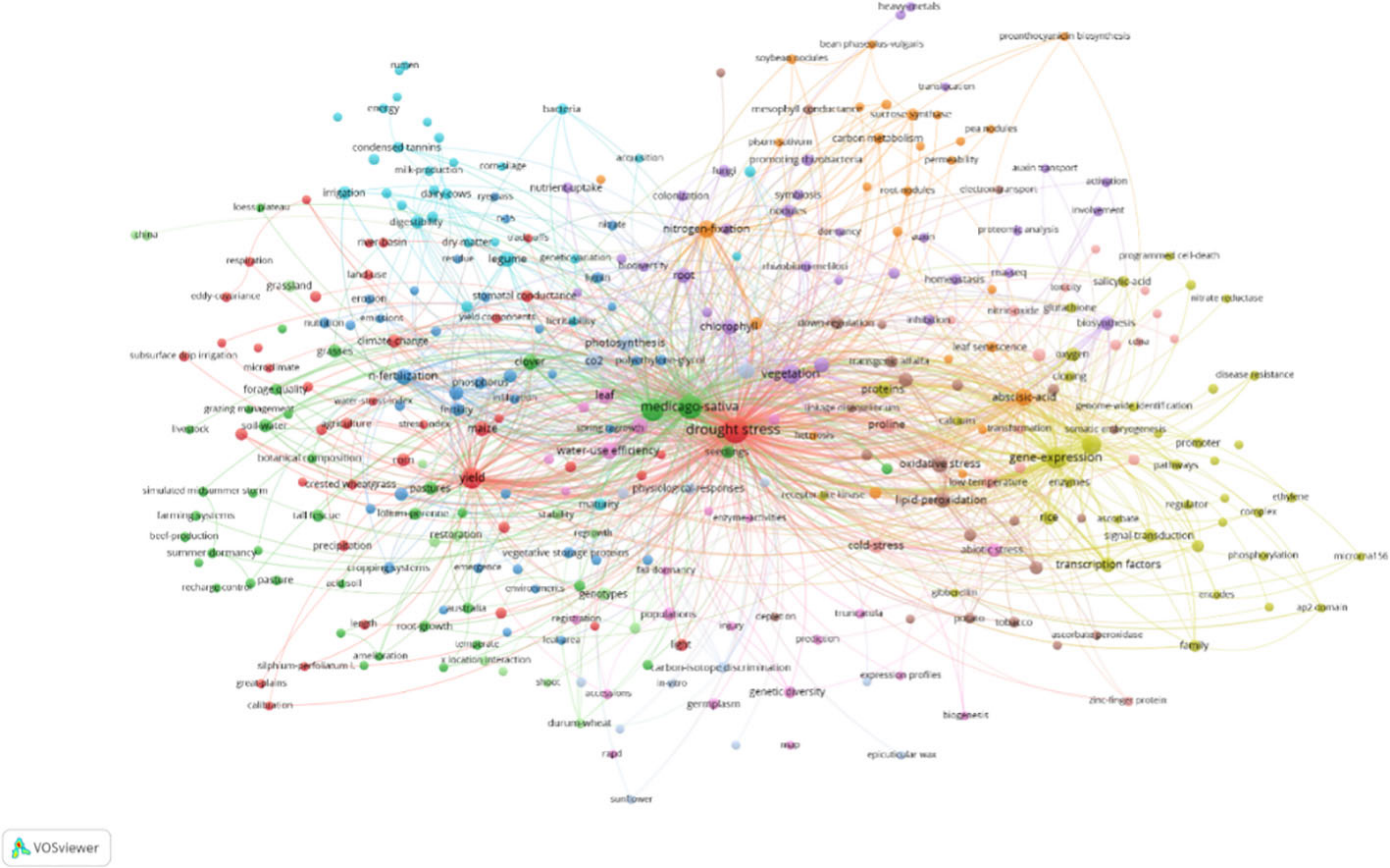
Knowledge domain map of the keywords co-occurrence network (frequency ≥3).

Keyword clustering analysis elucidates the relationships between keywords, revealing research hotspots. The visualization of this analysis in **Figure 6** identifies 12 core clusters in alfalfa drought resistance research: (1) Red cluster focuses on alfalfa yield, plant physiological responses to drought, and water resource management. (2) Green cluster delves into alfalfa growth and forage quality studies. (3) Blue cluster explores alfalfa seedling drought resistance using polyethylene glycol, weed control, and regeneration management techniques. (4) Yellow cluster investigates stress signal transduction related protein genes involving Ca^2+^ as a second messenger molecule. (5) Purple cluster covers inter-root microbial research. (6) Light blue cluster relates to alfalfa in animal husbandry production and irrigation management. (7) Orange cluster deals with symbiotic nitrogen fixation, crossbreeding, and related transcription factors. (8) Brown cluster examines antioxidant defense mechanisms during water stress. (9) Aqua red cluster focuses on genetic diversity analysis in agronomic traits. (10) Light brown cluster investigates osmotic regulation and related gene regulation during water deprivation. (11) Light green cluster examines drought-resistant morphological characteristics and environmental interactions. (12) Gray cluster studies photosynthesis and leaf morphology adaptations to drought stress.

A comprehensive analysis of the results in **Table 2** and **Figure 6** underscores two main research aspects: alfalfa’s response mechanisms to drought and drought-resistant technology research. These findings align with the conclusions derived from the analysis of the top 30 most influential papers, indicating a consistent focus within the field.

#### 3.4.2 Identification of research frontiers

**Figure 7:**
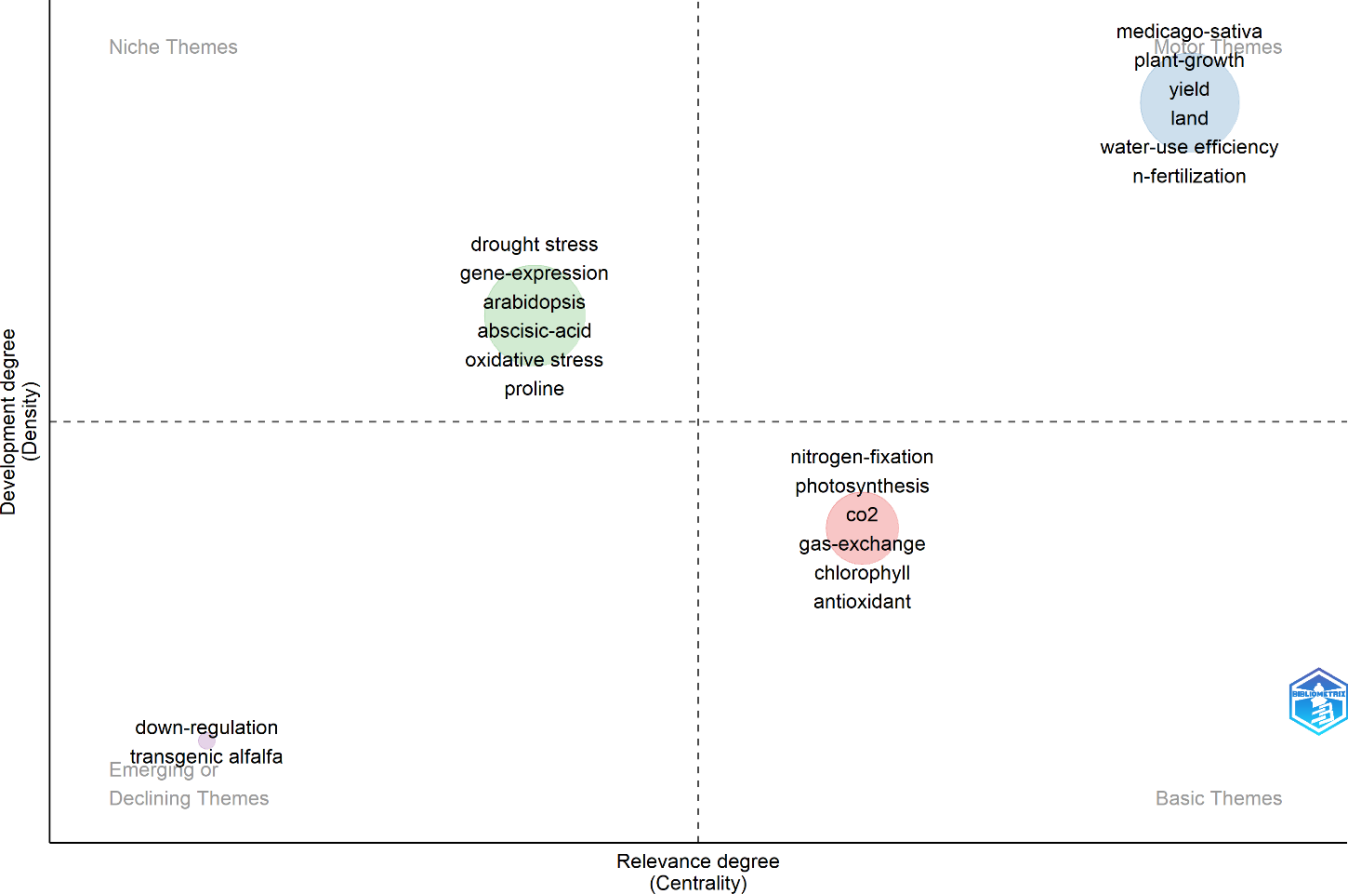
Thematic keywords on drought stress in *Medicago sativa* (frequency≥8).

**Figure 8:**
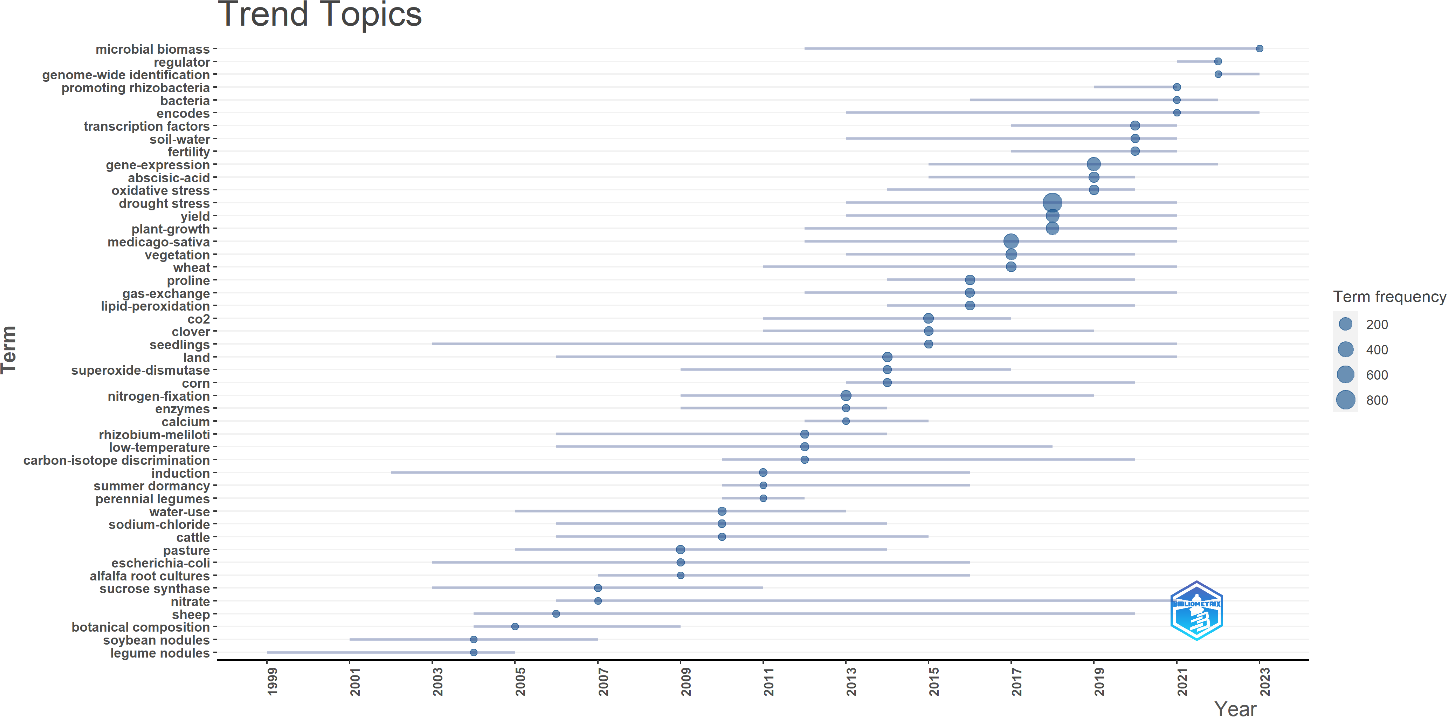
Keywords with the strongest occurrence bursts (frequency≥5).

**Figure 7** elucidates the categorization of research themes in the context of alfalfa drought resistance. The lower right quadrant, termed ‘Basic Themes,’ encompasses physiological and biochemical responses. These themes are pivotal to the field yet underdeveloped, signaling a need for further investigation. Contrarily, the upper right quadrant, ‘Motor Themes,’ comprises well-established and significant keywords, corroborating the findings presented in **Table 2**. ‘Niche Themes’ occupy the upper left quadrant, featuring keywords such as “drought stress,” “gene-expression,” “arabidopsis,” “abscisic-acid,” and “oxidative stress.” Although their significance and developmental extent are marginally lower than those in the ‘Motor Themes,’ their importance remains substantial. The lower left quadrant represents ‘Emerging or Declining Themes,’ with keywords like “down-regulation” and “transgenic alfalfa.” The inclusion of **Figure 8** in the analysis aids in further delineating these research frontiers.

The ‘Trend Topics’ section facilitates a visual analysis and projection of research focal points. A notable concentration of research activity, represented by the largest nodes, occurred between 2017 and 2019, particularly in areas such as “drought stress” and “gene-expression.” This trend highlights the significant attention devoted to these topics. Furthermore, the emergence of keywords like “regulator,” “bacteria,” and “gene-expression” up until 2022 suggests their potential as persistent research interests. Likewise, the appearance of terms such as “microbial biomass,” “genome-wide identification,” and “encodes” up to 2023 indicates their likelihood as future research directions. Cross-referencing **Figures 7** and **8** reveals that the themes in the lower left quadrant of **Figure 7** are gaining traction as emerging research areas. In conclusion, the current research frontiers in alfalfa drought resistance encompass gene expression studies, investigations into related microorganisms, and transgenic alfalfa research.

## 4 Discussion

Climate change, food shortages, water scarcity and population growth are some of the threatening challenges facing the world today. Drought stress (DS) is an ongoing challenge to agricultural crops and has been recognized as a serious constraint to global agricultural productivity, and its intensity and severity are expected to increase in the near future. Over the past two decades, the effects of drought stress on crop yield, growth and quality have increasingly become a major environmental issue (Wu et al., 2017) and a major limiting factor in alfalfa production (Ezura et al., 2015). Therefore, it is crucial to grasp the current status, frontiers and future research directions of the effects of drought stress on alfalfa in order to further improve alfalfa drought tolerance. In this paper, we collected and analyzed all the English academic publications on drought tolerance of alfalfa in the past 26 years for “article” and “review” by bibliometric analysis. Through bibliometric analysis, this study found that alfalfa drought stress research mainly includes two directions: response mechanism research and exploration of drought-resistant technology.

### 4.1 Drought resistance mechanisms

#### 4.1.1 Morphological, physiological and biochemical responses to drought resistance mechanisms

Under drought conditions, alfalfa undergoes significant morphological adaptations to sustain growth and development, particularly in its leaves and root system. Firstly, leaves, being the primary interface with the external environment, are highly responsive to environmental changes. Drought stress alters leaf morphology in alfalfa, affecting metrics such as leaf area ratio (LAR), specific leaf area (SLA), and leaf weight ratio (LWR). SLA significantly decreases with decreasing moisture content (Gu and Wan, 2021). These changes in SLA or LAR can be instrumental in identifying drought-resistant alfalfa varieties (Erice et al., 2010). Secondly, the root system, a vital component of the soil-plant-atmosphere continuum, exhibits a positive correlation with drought tolerance. It plays a crucial role in adapting to drought-stressed environments. Under drought stress, as soil moisture diminishes, there is an increase in both the volume and length of alfalfa’s root system, and a concurrent decrease in root diameter. This results in an expanded root-soil contact area, enhancing the plant’s capacity to absorb and utilize water from deeper soil layers (Zhu et al., 2008). Recent studies have demonstrated that larger leaves and robust roots during drought contribute to the accumulation of higher biomass yield (Prince et al., 2022). Moreover, the root-crown ratio in alfalfa is particularly sensitive to drought stress. An increase in this ratio under water stress conditions is indicative of a reduction in water consumption and an enhancement in water uptake (Erice et al., 2010), serving as a significant morphological index for assessing alfalfa’s drought resistance. The root-crown ratio stress index further reflects the drought resistance performance of alfalfa (Ma et al., 1993; Han et al., 2006).

In response to water deficits, alfalfa employs a range of physiological and biochemical strategies to mitigate drought stress. These responses can be categorized into five primary aspects.

**(1) Water metabolism:** Under drought conditions, there is a notable decrease in alfalfa’s leaf water potential and relative water content (RWC), along with an increase in water saturation deficit. Varieties with robust drought resistance exhibit a more pronounced decrease in leaf water potential, a lesser rise in water saturation deficit, and a smaller decline in RWC (Han et al., 2008; Ren and He, 2010), thus maintaining higher aboveground RWC and water use efficiency (WUE) (Buddhaboon et al., 2011; Anower et al., 2015). Furthermore, leaf surface cuticle waxes play a pivotal role in enhancing plant water use efficiency during water deficits by minimizing cuticle water loss (Samdur et al., 2003). It is observed that the wax content is more sensitive to drought treatments than the total wax content (Ni et al., 2012), and an increase in alkane content is particularly significant in improving drought tolerance, serving as a potential indicator for selecting drought-resistant alfalfa varieties (Guo et al., 2011; Ni et al., 2012). The introduction of chitosan seeds can effectively regulate the water use process and improve the yield and quality of alfalfa (Mustafa et al., 2022).
**(2) Photosynthesis**: Drought stress adversely affects chlorophyll biosynthesis while accelerating its catabolism, resulting in a substantial reduction in chlorophyll content and consequently inhibiting photosynthesis (Fang and Xiong, 2014). Drought conditions have been shown to decrease chlorophyll a and b, as well as photochemical efficiency in alfalfa, along with a significant increase in leaf MDA and proline contents (Fan et al., 2014). A decrease in leaf water potential to -2.8 MPa inhibits photosynthesis and reduces CO_2_ utilization due to stomatal closure (Irigoyen et al., 1992).
**(3) Compatible solutes and osmoregulation:** Research focusing on the accumulation of osmoregulatory substances in alfalfa under drought stress predominantly centers around organic osmoregulators such as proline, betaine, soluble protein (SP), and soluble sugar (SS) (Slama, 2013; Yang et al., 2013). These substances tend to increase with the duration and intensity of the drought (Yu et al., 2006; Li et al., 2007; Huo, 2010). Studies have indicated that drought-tolerant alfalfa cultivars accumulate higher levels of osmoregulatory substances compared to drought-sensitive varieties (Kang et al., 2011).
**(4) Antioxidant Defense:** Alfalfa’s adaptability to drought stress is closely linked to its antioxidant capacity. The activities of antioxidant enzymes (such as SOD, POD, and CAT) are significantly enhanced under drought stress, contributing to the plant’s increased resistance to drought (Han et al., 2005; Li et al., 2007; Huo, 2010; Quan et al., 2016). Studies have demonstrated that drought-resistant alfalfa cultivars exhibit lower electrolyte conductivity and MDA content under drought conditions, suggesting stronger resistance to oxidative stress (Hartung et al., 2001; Boldaji et al., 2011). Maghsoodi et al. confirmed a significant positive correlation between grass yield and peroxidase (POX), and a significant negative correlation with MDA, indicating that grass yield can serve as a marker for selecting drought tolerant varieties of alfalfa (Maghsoodi et al., 2017).
**(5) Plant endogenous (exogenous) hormones:** Abscisic acid (ABA) is a critical stress hormone playing a significant role in the plant’s response to drought stress (Hartung et al., 2001). ABA regulates various physiological processes, including stomatal closure and the modulation of leaf expansion and cell division rates (Schachtman and Goodger, 2008; Tardieu, 2013). Research has shown that the ABA content in alfalfa varies between roots and leaves under drought conditions and is influenced by the duration of the stress (Ren and He, 2010; Gamal et al., 2016). The plants that were not sensitive to ABA during germination showed stronger drought tolerance (Xu et al., 2024). Additionally, the accumulation of ABA in alfalfa under drought stress is related to the dynamics of storage proteins, gene expression, and the accumulation of certain osmoregulatory substances in the primary roots (Li et al., 2015).Overall, these physiological and biochemical responses collectively enhance alfalfa’s ability to withstand drought conditions, highlighting the complexity and adaptability of this crop in response to water stress.

#### 4.1.2 Molecular responses to drought resistance mechanisms

In recent years, owing to the extensive application of molecular biology technology (Huang et al., 2022; Zhao et al., 2022a; Zhao et al., 2022b), research on alfalfa drought resistance has progressed to the molecular level, such as identification of relevant stress genes, transcriptomics, metabolomics, and proteomics (Zhang et al., 2014; Li et al., 2021). The primary focus of this molecular investigation is the study of functional genes (proteins) associated with drought resistance in alfalfa. Transcription factors have emerged as key players in the response of alfalfa to drought stress (Zhao et al., 2022c). MicroRNA156 (miR156), for instance, regulates the Squamosa promoter binding protein (SPL) gene family, which acts as transcription factors that subsequently modulate the expression of downstream genes, thus regulating various plant growth and developmental networks (Cardon et al., 1999). Arshad and colleagues (Arshad et al., 2017) conducted experiments to explore the role of miR156d in alfalfa’s response to drought stress. Their findings revealed that, in comparison to the wild-type control (WT), alfalfa genotypes overexpressing miR156 (referred to as miR1560E) exhibited significantly higher drought tolerance. The miR1560E genotype not only displayed increased survival rates and reduced water loss but also maintained higher stomatal conductance, enhanced accumulation of compatible solutes (e.g., proline), and elevated levels of abscisic acid (ABA) and antioxidants during drought stress, when compared to the WT. Similarly, alfalfa plants with reduced expression of miR156-targeted SPL13 demonstrated decreased water loss, enhanced stomatal conductance, elevated chlorophyll content, and improved photosynthetic assimilation. These results suggest that miR156 enhances drought tolerance in alfalfa, partially by suppressing the expression of SPL13. Expression analysis of transcription factor genes related to the endoplasmic reticulum stress signaling pathway in alfalfa confirmed that DTT treatment reduced the functionality of bZIP60 and bZIP28 genes involved in the ER stress pathway. The most critical time for plants to tolerate drought (PEG) is the 8th hour after treatment, during which bZIP60 plays a more active role than bZIP28 in the stress pathway; the leaf tissues were more affected than the root tissues (Oğuz et al., 2022).

Transcriptomics reflect the transcriptional expression and regulation of genes in different genotypes across specific cells, tissues, or organisms under various adversities and developmental stages, thereby identifying candidate genes and revealing interactions among different gene regulatory pathways (Mutz et al., 2013; Palovaara et al., 2013). Feyissa et al. further demonstrated that the miR156/SPL13 module alleviates drought stress in alfalfa through tissue-dependent regulatory molecules and physiological processes (Feyissa et al., 2020). Feyissa et al. further demonstrated that the miR156/SPL13 module alleviates drought stress in alfalfa through tissue-dependent regulatory molecules and physiological processes (Wan et al., 2021). Feyissa et al. further demonstrated that the miR156/SPL13 module alleviates drought stress in alfalfa through tissue-dependent regulatory molecules and physiological processes (Fang et al., 2023). Moreover, the sensitivity of alfalfa seeds to ABA is heritable, and screening for ABA during seed germination can help select alfalfa lines with better drought tolerance (Xu et al., 2024).

Metabolomics studies aim to identify specific traits associated with stress tolerance by examining the molecular phenotypes of plants under abiotic stress. Feyissa and colleagues (Feyissa et al., 2019) further elucidated the regulation of drought stress in alfalfa by coordinating gene expression with metabolites and physiological strategies involving miR156/SPL13 and WD40–1/DFR. Modest levels of miR156 overexpression led to the suppression of SPL13 and an increase in WD40–1, thereby fine-tuning DFR expression to enhance anthocyanin biosynthesis. This, in conjunction with the accumulation of other stress relief metabolites and physiological responses, contributed to improved drought tolerance. Dominant classes of differential metabolites include amino acids, organic acids, sugars, and alkaloids, such as 6-gingerol, salicylic acid (SA), indole-3-acetic acid (IAA), gibberellin A4 (GA4), abscisic acid (ABA), trans cinnamic acid, sucrose, L-phenylalanine, L-tyrosine, succinic acid, and nicotinic acid, essential for drought stress tolerance in alfalfa (Wang et al., 2024).

Proteomics studies have increasingly reported on the response of alfalfa to drought stress. A total of drought stress-responsive proteins that play a role in a variety of cellular functions in the alfalfa root system were identified in the study by Rahman and colleagues (Rahman et al., 2016). These functions include energy metabolism, signaling pathways, antioxidant mechanisms, stress defense mechanisms, transcriptional and translational processes, regulation of reactive oxygen species (ROS), abscisic acid (ABA) biosynthesis, calcium signaling and storage processes. Ma et al. investigated the proteomic changes during the germination stage of Zhongmu NO.3 seeds under 200 mol·L^-1^ NaCl and 180 g·L^-1^ PEG stress, identifying 17 differentially abundant proteins (DAPs) mainly involved in defense responses, energy metabolism, protein synthesis and degradation, oxidative stress, and carbohydrate metabolism. Osmotic stimulation, used as a pretreatment, accelerates germination and improves the uniformity of seedling growth (Ma et al., 2017).

### 4.2 Drought-resistant technologies include breeding and cultivation

Another critical aspect of alfalfa drought resistance research involves the exploration of drought-resistant technologies, encompassing both breeding and cultivation techniques. Given the looming threat of global warming, drought stands as a major limiting factor for present and future alfalfa production, emphasizing the necessity of developing drought-resistant alfalfa varieties.

#### 4.2.1 Breeding techniques for drought resistance

Currently, conventional techniques continue to dominate the breeding of alfalfa varieties, with methods such as selective breeding and crossbreeding being the prevalent approaches. Simultaneously, molecular techniques have been progressively integrated into alfalfa breeding strategies. Selection breeding comprises various methods, including single selection, mixed selection, modified mixed selection, group selection, and rotational selection. For instance, Li et al (Li et al., 2011)successfully bred the drought- and cold-resistant Longmu 808 alfalfa by employing single-plant selection within the original population of Longmu 803 alfalfa, a process involving the selection of the best specimens while eliminating the least suitable.

Hybrid breeding, on the other hand, involves crossing individuals from different populations and genotypes to generate pure varieties through selection among their hybrid progeny. Notable Chinese registered varieties, such as Caoyuan No.1, Caoyuan No.2, and Gannong No.1, were bred through crosses between the tetraploid subsp. sativa and the diploid subsp. falcata (Shi et al., 2017). However, achieving a stable and mature variety through hybrid breeding requires multiple generations of backcrossing and faces challenges similar to standard crossbreeding.

An alternative approach is breeding based on male sterile lines, which offers a comprehensive method to produce genuine hybrids but is hampered by the difficulty of generating and maintaining stable male sterile lines. The CAU group employed a reverse genetic strategy, leveraging genome editing tools, to create genetically male sterile alfalfa and corresponding maintenance lines (Ye et al., 2022). Following ten generations of crossings between male sterile lines and maintenance lines, transgene-free male sterile lines were obtained, which can serve as parent materials for hybrid seed production.

Overcoming the challenge of cultivating purebred parents in alfalfa breeding is complicated due to its partial self-incompatibility and inbreeding suppression, whose molecular basis remains unclear. Molecular breeding, a contemporary approach, facilitates the rapid, stable, and directed creation of new varieties or species at the molecular level (Cai and Wang, 2003). As alfalfa genomics continues to advance, molecular marker technology can pinpoint drought-resistant genes or genes closely associated with drought resistance in alfalfa. This enables the swift identification of drought-tolerant varieties, shortening the breeding cycle and, in theory, reducing costs (Charles and Yu, 2018). However, its practical implementation hinges on the accuracy of marker data and thorough validation, particularly considering alfalfa’s tetraploid nature and complex genetic characteristics.

In tandem with the progress in genetic engineering, transgenic technology has emerged as a novel avenue for enhancing alfalfa’s drought resistance and improving variety traits. Zhang and colleagues (Zhang et al., 2005) reported the successful activation of alfalfa’s waxy production and the conferment of drought tolerance through the introduction of the WXP1 gene from the model legume *Tribulus terrestris*. This resulted in a notable increase in leaf wax accumulation and enhanced drought tolerance in transgenic alfalfa.

Additionally, Nie et al. (Nie et al., 2017) observed increased proline accumulation in alfalfa through the overexpression of the AmDHN gene, a change that potentially induces the expression of genes related to the proline synthesis pathway, ultimately bolstering drought tolerance. Fehér-Juhász and colleagues (Fehér-Juhász et al., 2013) demonstrated that ectopic expression of the GsWRKY20 gene led to increased osmoregulatory substance accumulation, improved water-holding capacity, decreased membrane permeability, reduced MDA content, and enhanced drought tolerance in transgenic alfalfa. Mckersie and colleagues (McKersie et al., 2000) transplanted the Mn-SOD gene from blue snowy tobacco into alfalfa using *Agrobacterium*, with field experiments confirming the significant enhancement of cold and drought resistance in transgenic alfalfa plants.

These advancements in breeding and biotechnological approaches hold promise for mitigating the impact of drought on alfalfa production, offering potential solutions for sustaining alfalfa cultivation in the face of global climate challenges.

#### 4.2.2 Cultivation techniques for drought resistance

Cultivation techniques aimed at enhancing water stress resistance in alfalfa encompass several strategies. The first strategy involves water conservation and drought resistance, which includes techniques such as seed triggering (Li et al., 2011; Antoniou et al., 2020), the utilization of irrigation systems (Annicchiarico et al., 2012; Ismail and Almarshadi, 2013; Huang et al., 2018), and adjustments in the timing of product harvest (Liu et al., 2018). Furthermore, the traditional application of organic fertilizers and other materials like sludge serves to protect alfalfa nodules from oxidative stress (Antolín et al., 2010). The application of plant growth-regulating substances, such as melatonin (Cardon et al., 1999), and the utilization of beneficial bacterial strains, including cytokinin-engineered bacteria (Xu et al., 2012), have been shown to improve drought resistance in alfalfa.

Cultivation of alfalfa is not limited to field management, and the establishment of a suitable alfalfa standardized growth and development patterns and designing ideal cultivation practices for different ecological zones is imperative. Alfalfa can be cultivated either as a pure stand or in rotation or intercropping systems, and a rational model necessitates a balanced relationship with companion crops. This presents both advantages and challenges (Buddhaboon et al., 2011; Grieder et al., 2021; Peng et al., 2022; Xu et al., 2022; Wang et al., 2023).

To enhance drought resistance, the cultivation process incorporates selecting appropriate alfalfa varieties and applying effective nutrient management, weed control, and pest management. These elements are pivotal in achieving optimal harvests and are crucial for sustaining alfalfa production under drought conditions (Putnam, 2021; Undersander, 2021; Graham et al., 2022; Wang et al., 2022; Yin et al., 2022). Additionally, mechanization of the entire production process is essential for alfalfa cultivation (Putnam, 2021). This requires alfalfa plants with an upright growth habit and robust resistance to lodging.

## 5 Conclusions

In this study, using alfalfa or *Medicago sativa* etc. as keywords, 1141 documents were collected in Web of Science database and 1081 were screened for bibliometric study. The findings revealed a steady increase in alfalfa drought tolerance research through the publication of articles in recent years. China contributed the most articles in this field, with 385 publications, followed by the United States and Australia. Notably, the journal “Plant Physiology” published the highest number of articles related to alfalfa drought tolerance research, with a total of 37 articles and 2082 citations. Through an analysis of the top 30 cited literature and keywords, the study identified the key developments in alfalfa drought stress research. The current research hotspots center around understanding the mechanisms by which alfalfa responds to water deficit, encompassing morphology, physiology, biochemistry, and molecular responses. Additionally, there is a growing emphasis on enhancing drought-related technologies, including drought-resistant breeding and optimization of cultivation techniques. Among these hotspots, research on the physiological, biochemical, and molecular response mechanisms of alfalfa, as well as investigations into transgenic alfalfa and nutrient management (such as microbial fertilizers), are expected to remain significant and likely become even more prominent in future research endeavors. The primary goal of this study is to provide theoretical guidance for alfalfa drought tolerance research and to contribute to the enhancement of alfalfa crop yield.

## Data availability statement

The original contributions presented in the study are included in the article/**Supplementary Material**. Further inquiries can be directed to the corresponding author.

## Author contributions

ZZ: Formal analysis, Methodology, Software, Writing – original draft. JL: Formal analysis, Visualization, Writing – review & editing. YG: Visualization, Writing – review & editing. XW: Formal analysis, Visualization, Writing – review & editing. RW: Supervision, Visualization, Writing – review & editing. HH: Supervision, Visualization, Writing – review & editing. YZ: Supervision, Visualization, Writing – review & editing. LZ: Formal analysis, Supervision, Visualization, Writing – review & editing. PW: Formal analysis, Funding acquisition, Methodology, Supervision, Visualization, Writing – review & editing.
