# Supplementary material for: Research on drought stress in Medicago sativa L. from 1998 to 2023: a bibliometric analysis
Source: Front Plant Sci. 2024 May 30;15:1406256. doi: 10.3389/fpls.2024.1406256 (PMC11169798; doi:10.3389/fpls.2024.1406256)
Supplement: Supplementary file 1 [file DataSheet_1.pdf]

Table.S1 Top 20 most productive countries/regions on drought stress in *Medicago sativa* researches ranked by total number of publications. Note: a-Total Publications. b-Percentage (%).c-Total Citations. d-Avg. Citations. e-Links.f-Total Link Strength.

Rank	Country	Cluster	TP[a]	P[b]	TC[c]	CPP[d]	L[e]	TLS[f]
1	China	6	385	35.62%	7229	18.78	26	99
2	USA	9	213	19.70%	6240	29.30	39	104
3	Australia	7	116	10.73%	2601	22.42	20	55
4	Spain	1	76	7.03%	2596	34.16	23	60
5	Canada	5	59	5.46%	1320	22.37	15	34
6	Iran	10	51	4.72%	676	13.25	13	18
7	France	1	40	3.70%	1366	34.15	18	46
8	Italy	1	36	3.33%	928	25.78	13	25
9	Germany	4	27	2.50%	688	25.48	19	24
10	Morocco	1	27	2.50%	996	36.89	11	30
11	South Korea	6	23	2.13%	1297	56.39	7	20
12	New Zealand	3	21	1.94%	430	20.48	7	13
13	Tunisia	1	21	1.94%	357	17.00	16	22
14	Pakistan	8	16	1.48%	378	23.63	12	20
15	India	8	15	1.39%	296	19.73	6	9
16	Turkey	6	14	1.30%	284	20.29	10	13
17	Greece	4	13	1.20%	201	15.46	2	2
18	Hungary	4	12	1.11%	527	43.92	7	10
19	Mexico	7	12	1.11%	201	16.75	5	13
20	Poland	8	11	1.02%	156	14.18	1	1

Table.S2 Top 20 institutions with the most publications on drought stress in *Medicago sativa.* Note: a-Total Publications. b-Percentage (%).e-Links. f-Total Link Strength

Rank	Institution	Country	Cluster	TP[a]	P[b]	L[e]	TLS[f]
1	chinese acad agr sci	China	5	64	5.92%	85	2856.55
2	chinese acad sci	China	4	59	5.46%	87	2670.85
3	lanzhou univ	China	7	56	5.18%	87	1804.68
4	china agr univ	China	5	38	3.52%	83	1413.70
5	agr & agri food canada	Canada	6	35	3.24%	86	1952.19
6	northwest a&f univ	China	4	35	3.24%	86	1349.24
7	usda ars	USA	1	33	3.05%	86	1249.84
8	gansu agr univ	China	7	28	2.59%	85	713.80
9	univ chinese acad sci	China	4	27	2.50%	85	1412.15
10	univ navarra	Spain	2	27	2.50%	85	1095.73
11	univ western	Australia	3	25	2.31%	84	1150.34

	australia						
12	csic	Spain	2	21	1.94%	86	847.82
13	nanjing agr univ	China	8	21	1.94%	83	614.78
14	inner mongolia agr univ	China	4	20	1.85%	81	672.87
15	northeast agr univ	China	4	19	1.76%	78	396.87
16	univ publ navarra	Spain	2	17	1.57%	87	805.32
17	utah state univ	USA	1	15	1.39%	86	472.85
18	samuel roberts noble fdn inc	New zealand	1	14	1.30%	85	501.81
19	univ calif davis	California	1	14	1.30%	86	585.94
20	univ barcelona	Spain	2	13	1.20%	85	658.22

Table.S3 Top 20 most cited journals on drought stress in *Medicago sativa.* Note: a-Total Publications. b-Percentage (%). IF-Impact Factors. c-Co Citations. d-Avg. citations. e-Links.f-Total Link Strength.

Rank	Journal title	Country	Cluster	IF	CC[c]	L[e]	TLS[f]
1	plant physiol	USA	2	7.4	2268	259	2014.17
2	j exp bot	England	4	6.9	1313	259	1239.22
3	crop sci	USA	3	6.9	1272	260	1090.68
4	front plant sci	Switzerland	2	4.9	1009	258	946.09
5	plant soil	Netherlands	1	5.6	950	260	884.97
6	plant j	England	2	2.1	855	255	811.63
7	agron j	USA	1	7.2	844	257	734.79
8	plant cell environ	England	4	7.3	780	260	741.81
9	plant cell	USA	2	12	745	237	702.19
10	physiol plantarum	Denmark	4	1.37	742	258	707.43
11	aust j agr res	Australia	3	6.4	726	253	572.41
12	new phytol	England	4	9.4	659	259	635.80
13	j plant physiol	Gernamy	4	4.3	632	255	606.24
14	plant sci	Ireland	2	5.2	612	256	594.54
15	plant physiol bioch	Franch	2	6.5	599	255	578.61
16	plos one	USA	2	3.7	590	260	573.29
17	aust j exp agr	Australia	3	1.621	588	240	451.38
18	agr water manage	Netherlands	1	6.7	574	244	484.60
19	planta	Gernamy	2	5.2	559	252	542.75
20	p natl acad sci usa	USA	2	11.1	556	259	536.12
